# Brief Screening Instruments for Alcoholism

**Published:** 1997

**Authors:** Cheryl J. Cherpitel

**Affiliations:** Cheryl J. Cherpitel, Dr.P.H., is a senior scientist in the Alcohol Research Group at the Public Health Institute, Berkeley, California

Numerous studies have demonstrated that many problem drinkers (i.e., people who fulfill the diagnoses of harmful drinking, alcohol abuse, or alcohol dependence) can benefit from brief physician interventions at the time of a clinic visit or from referral for alcoholism treatment (Buchsbaum 1994). To identify problem drinkers in clinical and primary care settings, researchers have developed several screening instruments, most of them short questionnaires that can be administered by a physician or self-administered by the patient. This article reviews some of these instruments as well as their effectiveness in identifying problem drinkers in primary care settings, such as emergency rooms (ER’s).

## Assessing the Validity of Screening Instruments

To assess the validity of screening instruments, researchers have evaluated the instruments’ ability to identify problem drinkers who fulfill the diagnostic criteria for alcohol use disorders as listed in the *International Classification of Diseases, Tenth Edition* (ICD–10) (World Health Organization 1990) or the *Diagnostic and Statistical Manual of Mental Disorders, Fourth Edition* (DSM–IV) (American Psychiatric Association 1994). These analyses have found that various instruments differ in their validity. One factor affecting validity is whether the instrument assesses drinking problems that have occurred at any time in the patient’s life or only recent drinking problems (i.e., usually within the past 12 months). In most clinical settings, the diagnostic focus is on identifying current alcohol problems; accordingly, instruments that assess current or recent drinking problems would possess greater validity in these settings compared with instruments that assess lifetime drinking problems.

Two important measures of a screening instrument’s validity in clinical settings are its sensitivity and specificity. Sensitivity is defined as the percentage of all patients with a condition (e.g., alcoholism) who are correctly identified by the instrument as having the condition. Thus, a sensitivity of 80 percent indicates that an instrument correctly identifies 80 percent of all alcoholics in a sample. Specificity is defined as the percentage of all patients without a condition who are correctly identified as not having the condition. For example, a specificity of 90 percent indicates that the instrument correctly identifies 90 percent of all nonalcoholics in a sample. As the sensitivity of an instrument increases, its specificity usually decreases. In practice settings, sensitivity generally is considered more relevant than specificity, because it is important to identify all potential problem drinkers, even if some nonproblem drinkers may be falsely identified as well.

## Current Screening Instruments

If physicians are to accept and use a screening instrument to identify problem-drinking patients in their practices, the instrument must not only be sensitive and reasonably specific but also brief and easy to use and score. Several currently available screening instruments meet these criteria, including the Quantity/Frequency Questions, the CAGE, the brief Michigan Alcoholism Screening Test (BMAST), the Alcohol Use Disorders Identification Test (AUDIT), and the TWEAK.

The Quantity/Frequency Questions—developed by the National Institute on Alcohol Abuse and Alcoholism (1995) (see box below) and currently used by the majority of physicians—include three questions that assess the drinker’s frequency and level of alcohol consumption. Drinkers whose alcohol consumption exceeds the recommended levels may be at risk for alcohol-related problems. These people should be assessed further to determine whether they have experienced any alcohol problems or are alcohol dependent.

**Determining Quantity and Frequency of Alcohol Consumption**

- On average, how many days per week do you drink alcohol?
- On a typical day when you drink, how many drinks do you have?
- What is the maximum number of drinks you have had on any given occasion during the past month?

NOTE: Men who drink more than ^14^ drinks per week or more than ^4^ drinks per occasion and women who drink more than ^7^ drinks per week or more than ^3^ drinks per occasion may be at risk for alcohol-related problems. These people should be assessed further to determine the nature and extent of their alcohol-related problems.SOURCE: Adapted from National Institute on Alcohol Abuse and Alcoholism 1995.

The CAGE (Ewing 1984) (see box on right) is a four-question instrument that has been used primarily in clinical settings to identify people who have ever been alcohol dependent. A positive response to two or more of the four items is generally considered the cutoff value, indicating a potential alcohol problem. Using this cutoff value, the CAGE’s sensitivity in various populations ranges from 61 to 100 percent, and its specificity ranges from 77 to 96 percent (Cherpitel 1997*b*). The CAGE questions also can be included in questionnaires that assess various health-related behaviors (e.g., smoking, weight, and exercise patterns), such as the Health Screening Survey and the PRIME-MD.

**CAGE: An Alcoholism Screening Test**

- Have you ever felt you should **C**UT down on your drinking?
- Have people **A**NNOYED you by criticizing your drinking?
- Have you ever felt bad or **G**UILTY about your drinking?
- Have you ever had a drink first thing in the morning to steady your nerves or to get rid of a hangover (i.e., as an **E**YE-OPENER)?

NOTE: Two positive responses to these questions are considered a positive test and indicate that further assessment is warranted.SOURCE: Adapted from Ewing 1984.

The BMAST (Pokorny et al. 1972)—a 10-question subset of the original 25-item Michigan Alcoholism Screening Test (MAST) (Selzer 1971)—has been found to be reliable and sensitive in both clinical and nonclinical settings. When scored according to the MAST, with a cutoff value of six points, the results of the BMAST are highly correlated with those of the MAST (Pokorny et al. 1972). The overall sensitivity of the BMAST ranges from 30 to 78 percent, and the specificity ranges from 80 to 99 percent (Cherpitel 1997*b*).

The 10-question AUDIT was developed by the World Health Organization to identify problem drinkers in primary care settings (Saunders et al. 1993). In a six-nation validation trial, the AUDIT demonstrated high sensitivity and specificity in identifying heavy drinkers when a cutoff value of eight points was used (each question can score zero to four points) (Saunders et al. 1993). A recent review of the AUDIT’s performance found that the test’s sensitivity ranged from 38 to 94 percent and its specificity ranged from 66 to 90 percent (Allen et al. 1997).

The TWEAK (see box at top right) is a relatively new screening instrument that initially was designed to identify “at-risk” drinking in pregnant women (Russell et al. 1994). Of its five questions, two were taken from the CAGE, two were taken from the MAST, and the fifth question was newly added. The test is scored on a seven-point scale, two points each for a positive response to either one of the first two questions and one point for each of the last three questions. With a cutoff value of two points, the TWEAK has a sensitivity of 79 percent and a specificity of 83 percent for identifying pregnant women who consume 1 or more ounces of absolute alcohol per day.^1^ Using a weighted cutoff value of three points, the sensitivity of the TWEAK ranges from 70 to 90 percent and the specificity from 75 to 80 percent (Cherpitel 1997*b*).

**TWEAK: An Alcoholism Screening Test Developed for Women**

- **T**OLERANCE: How many drinks can you hold?
- Have close friends or relatives **W**ORRIED or complained about your drinking in the past year?
- **E**YE OPENER: Do you sometimes take a drink in the morning when you first get up?
- **A**MNESIA: Has a friend or family member ever told you about things you said or did while you were drinking that you could not remember?
- Do you sometimes feel the need to C(**K**)UT DOWN on your drinking?

NOTE: The TWEAK test is scored based on a seven-point scale, two points each for a positive response to either one of the first two questions and one point for each of the last three questions. A total score of two or more points indicates that the woman is likely to have an alcohol problem.SOURCE: Adapted from Russell et al. 1994.

## Comparison of Screening Instruments

Many screening instruments (e.g., the CAGE) have been developed for and tested on known alcoholics, primarily white males. Other instruments have been designed to identify problem drinkers who have not yet become alcohol dependent (e.g., the AUDIT) or to be more sensitive to problem drinking among women (e.g., the TWEAK). Few studies, however, have compared the performance of these instruments in men and women, various ethnic groups, or patients in primary care settings (including ER’s) who have a high prevalence of problem drinking.

A study at the University of Mississippi Medical Center in Jackson, Mississippi, compared the sensitivity and specificity of the CAGE, BMAST, AUDIT, and TWEAK in identifying patients who had fulfilled the ICD–10 diagnostic criteria for alcohol dependence and/or harmful drinking during the previous 12 months (Cherpitel 1995*b*,*c*). The study involved a representative sample of 1,330 ER patients (i.e., the Mississippi sample). All participants were current drinkers (i.e., they reported drinking during the past 12 months), because current drinkers were considered most likely to be at risk for alcohol problems. All of the questions included in the AUDIT and four of the five items included in the TWEAK pertain to the person’s drinking habits over the previous 12 months. In contrast, the remaining question on the TWEAK and all items on the CAGE and BMAST refer to any time in a person’s life. Thus, study participants who responded positively to one of these lifetime-based items were then asked whether the episode had occurred in the past 12 months.

The study separately compared the instruments’ performances in men and women and in African-American and Caucasian patients. The results can be summarized as follows (Cherpitel 1995*a*,*b*,*c*):

- For the entire study group, the AUDIT and TWEAK demonstrated greater sensitivity (85 and 87 percent, respectively) than did the CAGE (75 percent) or the BMAST (31 percent).
- All four instruments were more sensitive for men than for women.
- Although the AUDIT and TWEAK were more sensitive for women compared with the CAGE and BMAST, the sensitivity of both tests among women was much lower than among men. The sensitivity of the AUDIT was 72 percent for women and 93 percent for men, and the sensitivity of the TWEAK was 74 percent for women and 94 percent for men.
- The AUDIT and TWEAK exhibited equal sensitivity in African-Americans (88 and 86 percent, respectively), whereas the TWEAK was more sensitive than the AUDIT among Caucasians (90 and 77 percent, respectively).
- The sensitivity of the AUDIT and TWEAK did not differ between African-American and Caucasian men. For women, however, the AUDIT was more sensitive among African-Americans (71 percent), whereas the TWEAK was more sensitive among Caucasians (87 percent).
- When the cutoff values were lowered for the CAGE, AUDIT, and TWEAK, the instruments’ sensitivity among women improved, with no concurrent substantial loss of specificity. Among men, however, a lowering of the cutoff values did not improve sensitivity, but greatly reduced specificity.
- Lower cutoff values did not significantly improve instrument performance (i.e., sensitivity or specificity) for either African-Americans or Caucasians as a whole.
- All instruments were less sensitive when the analysis included patients with a diagnosis of either harmful drinking or alcohol dependence with harmful drinking than when the analysis included only patients with a diagnosis of alcohol dependence alone.

## The Rapid Alcohol Problems Screen (RAPS)

As indicated by the Mississippi study, the CAGE, BMAST, AUDIT, and TWEAK varied significantly in their performance across gender and ethnic subgroups. Moreover, the tests performed particularly poorly among women, especially African-American women. To determine a set of questions that would exhibit the highest sensitivity in this population while maintaining good specificity, items from all four instruments were evaluated independently. This analysis identified a new five-item instrument called the Rapid Alcohol Problems Screen (RAPS) (see box below), which, when using a cutoff value of one point, outperformed all other instruments in all subgroups (Cherpitel 1995*d*). Of the five questions included in the RAPS, two were derived from the TWEAK, two from the AUDIT, and one from the BMAST. Sensitivity for the RAPS was 93 percent for men and 84 percent for women; specificity ranged from 82 percent for women to 75 percent for men (Cherpitel 1995*d*).

**The Rapid Alcohol Problems Screen (RAPS)**

- Do you sometimes take a drink in the morning when you first get up?
- During the past year, has a friend or family member ever told you about things you said or did while you were drinking that you could not remember?
- During the past year, have you had a feeling of guilt or remorse after drinking?
- During the past year, have you failed to do what was normally expected of you because of drinking?
- During the past year, have you lost friends or girlfriends or boyfriends because of drinking?

NOTE: A positive answer to one of the questions is considered a positive test.SOURCE: Adapted from Cherpitel 1995*d*.

The validity of the RAPS was assessed in a second sample of 1,429 ER patients recruited at the Santa Clara Valley Medical Center in San Jose, California (i.e., the California sample) (Cherpitel in press). The subjects included men and women of African-American, Caucasian, and Hispanic ethnicity. The study determined the sensitivity and specificity of the RAPS in identifying current drinkers who had fulfilled the ICD–10 and/or DSM–IV criteria for alcohol dependence during the past 12 months. The sensitivity and specificity of the RAPS were then compared with those of the CAGE, BMAST, AUDIT, and TWEAK.

These analyses found that for women from all three ethnic groups, the sensitivity of the RAPS exceeded that of the other screening instruments. Thus, the sensitivity was 93 percent for African-American women, 92 percent for Hispanic women, and 90 percent for Caucasian women. The specificity ranged from 79 percent for African-American and Caucasian women to 85 percent for Hispanic women (Cherpitel in press). Among men, however, both the sensitivity (95 percent) and specificity (71 percent) of the RAPS were not quite as high as the values obtained with the AUDIT (97 and 74 percent, respectively). This finding was true for all three ethnic groups. The performance of the RAPS also was compared among participants of the same ethnicity in the Mississippi and California studies (Cherpitel 1997*a*). In this analysis the RAPS demonstrated greater sensitivity in African-American patients (both women and men) from the Mississippi study than from the California study. Conversely, among Caucasian patients, the RAPS was more sensitive for the California sample than for the Mississippi sample. The reasons for these differences are still unclear. However, the questions included in the RAPS were optimized to detect problem drinkers in the Mississippi sample, which included 85 percent African-American patients. Therefore, it appears plausible that the instrument performed best among the African-Americans in that sample.

## Conclusions

Short screening instruments, such as the CAGE, BMAST, AUDIT, and TWEAK, can be useful tools in identifying problem-drinking patterns. These instruments vary significantly, however, in their performance among different population subgroups. The RAPS was developed to improve the sensitivity and specificity of the existing instruments. In two studies the RAPS outperformed the other screening instruments in several population subgroups.

The findings of neither the Mississippi nor the California study can be generalized to other ER or primary care populations. Nevertheless, the data suggest that the RAPS may be a promising screening instrument for identifying problem drinkers across ethnic and gender subgroups and in various regions of the country. Because of its brevity and ease of scoring (e.g., patients need not be asked additional items after screening positive on any one of the five items), the RAPS may be particularly appealing for use in clinical settings. Further research is needed, however, to compare the performance of the RAPS with that of other screening instruments across demographic subgroups in a variety of clinical settings.

## Footnotes

1 One ounce of pure alcohol corresponds to approximately two drinks.
